# Comparison of Gender Differences in Intracerebral Hemorrhage in a Multi-Ethnic Asian Population

**DOI:** 10.1371/journal.pone.0152945

**Published:** 2016-04-06

**Authors:** Justin T. Hsieh, Beng Ti Ang, Yew Poh Ng, John C. Allen, Nicolas K. K. King

**Affiliations:** 1 School of Medicine, Duke-National University of Singapore Medical School, Singapore, Singapore; 2 Department of Neurosurgery, National Neuroscience Institute, Singapore, Singapore; 3 Center for Quantitative Medicine, Duke-National University of Singapore Medical School, Singapore, Singapore; National Cheng Kung University, TAIWAN

## Abstract

**Background:**

Intracerebral hemorrhage (ICH) accounts for 10–15% of all first time strokes and with incidence twice as high in the Asian compared to Western population. This study aims to investigate gender differences in ICH patient outcomes in a multi-ethnic Asian population.

**Method:**

Data for 1,192 patients admitted for ICH were collected over a four-year period. Multivariate logistic regression was used to identify independent predictors and odds ratios were computed for 30-day mortality and Glasgow Outcome Scale (GOS) comparing males and females.

**Result:**

Males suffered ICH at a younger age than females (62.2 ± 13.2 years vs. 66.3 ± 15.3 years; P<0.001). The occurrence of ICH was higher among males than females at all ages until 80 years old, beyond which the trend was reversed. Females exhibited increased severity on admission as measured by Glasgow Coma Scale compared to males (10.9 ± 4.03 vs. 11.4 ± 4.04; P = 0.030). No difference was found in 30-day mortality between females and males (F: 30.5% [155/508] vs. M: 27.0% [186/688]), with unadjusted and adjusted odds ratio (F/M) of 1.19 (P = 0.188) and 1.21 (P = 0.300). At discharge, there was a non-statistically significant but potentially clinically relevant morbidity difference between the genders as measured by GOS (dichotomized GOS of 4–5: F: 23.7% [119/503] vs. M: 28.7% [194/677]), with unadjusted and adjusted odds ratio (F/M) of 0.77 (P = 0.055) and 0.87 (P = 0.434).

**Conclusion:**

In our multi-ethnic Asian population, males developed ICH at a younger age and were more susceptible to ICH than women at all ages other than the beyond 80-year old age group. In contrast to the Western population, neurological status of female ICH patients at admission was poorer and their 30-day mortality was not reduced. Although the study was not powered to detect significance, female showed a trend toward worse 30-day morbidity at discharge.

## Introduction

Intracerebral hemorrhage (ICH) accounts for 10–15% of all first time strokes [1, 2]. It is associated with a one-month mortality of 40% and significant morbidity as compared to other types of stroke [3, 4]. There have been various studies looking into the epidemiology of such a devastating disease, but the effect of gender on incidence and mortality is not well characterized. Research findings from different parts of the world have demonstrated that women may have a lower incidence of ICH [5–7], less perihematomal edema, and lower mortality [8–11]. In particular, it has been suggested that old age interact with female gender to worsen outcome [10].

The incidence of ICH amongst Asians has been found to be 51.8 per 100,000 person-years, which is two-fold higher than in other parts of the world [4]. Therefore, it is important that we gain a better understanding of gender differences in this population, data of which has been very scant, particularly for the Southeast Asian group. In this retrospective study, we reviewed the epidemiology and clinical presentation of ICH in Singapore. We investigated the incidence of ICH relative to gender differences, age, ethnicity, clinical presentation, radiological findings, and cerebrovascular risk factors. Further, we compared morbidity and mortality in ICH patients according to gender.

## Methods

The Singapore Centralized Institutional Review Board (CIRB) of SingHealth granted a waiver of informed consent for this retrospective observational study. The decision was based on an understanding that the study posed minimal risk and would not adversely affect the rights and welfares of the research subjects.

### Study population

With Singapore CIRB of SingHealth approval, data were collected on all consecutive patients admitted to the National Neuroscience Institute during the 4-year period July 2004 to July 2008. A total of 1196 patients were admitted during this time period, 688 (58%) of whom were male. The study population was multi-ethnic including Chinese, Malay, Indian, and others. To protect patients’ right to privacy, all patient records were anonymized and de-identified prior to data analysis.

### Interventions

All patients were treated in accordance with the AHA/ASA guidelines on intracerebral hemorrhage in our institution.

### Data collection and computation

All clinical data on the acute presentation (Glasgow Coma Scale and blood pressure) were taken from information obtained at the initial evaluation in the emergency department. The initial head computed tomography (CT) images and reports were used to derive radiological information, including presence of intraventricular hemorrhage (IVH), presence of hydrocephalus (as reported by the duty radiologist), location of ICH and ICH clot size (using the ABC/2 method). Patient demographic data, medical history (background cerebrovascular risk factors), and in-hospital intervention were extracted from medical records. Background cerebrovascular risk factors included smoking history, hypertension, diabetes, hyperlipidemia, statin, warfarin and anti-platelet use. Glasgow Coma Scale (GCS) was used to assess severity of morbidity on admission. Mild GCS impairment was defined as GCS >12, moderate impairment as 9–12 and severe as ≤ 8. Thirty-day mortality rates were obtained from data held on a centrally updated online electronic patient record. Glasgow Outcome Scale (GOS) was collected on discharge. GOS of 5 denotes low disability, 4 moderate disability, 3 severe disability, 2 persistently vegetative state, and 1 denotes death.

### Statistical analysis

Patient demographics and clinical characteristics were summarized as mean ± standard deviation for continuous data, and frequency counts and percentages for categorical data. Patient demographic and clinical characteristic variables were compared using a two-sample t-test in the case of continuous variables and a chi-square test for categorical variables. Stepwise logistic regression (SLE = SLS = 0.05) on 30-day mortality (Y/N) and GOS (1–3 vs 4–5) was used to identify significant confounders. In the stepwise analysis, gender was forced into the model and significant confounders among baseline variables were identified. These confounders included GCS on admission, warfarin, antiplatelet use, fixed pupils on presentation, clot location, hydrocephalus, age and volume of clot. These were included in a multivariate logistic regression analysis to assess the independent effects of gender on 30-day mortality and GOS following intracranial hemorrhage. Statistical significance was declared at p <0.05. All analyses were performed using SAS V9.2 (SAS, Inc, Cary, NC, USA)

## Results

A total of 1196 patients were admitted to our center from July 2004 to July 2008 for ICH. 688 (58%) were male. Patient demographics by gender are summarized in Table 1 and clinical characteristics at admission in Table 2. The study population was multi-ethnic and comprised of Chinese, Malay, Indian and others. Ethnicity distributions between genders did not differ significantly. Males suffer ICH at a younger age with 62.2 years for males versus 66.3 years for females (P<0.001). ICH occurrence was proportionally higher in males than females for all age groups considered, except for the >80 years old group, for which the trend was reversed (P<0.0001).

**Table 1:** Demographics of intracranial hemorrhage patients by gender. Males developed ICH at a younger age than females (62.2 ± 13.2 vs. 66.3 ± 15.3; P<0.001). Males had a higher occurrence for ICH at all age groups up to 80 years old, beyond which there was a higher occurrence in females. Gender did not interact with ethnicity to cause a difference in ICH.

	Male (n = 688)	Female (n = 508)	P-value^1^
Ethnic group, *n* (*%*)			
Chinese	572 (83.1)	423 (83.6)	0.991
Malay	75 (10.9)	55 (10.9)	
Indian	23 (3.3)	16 (3.2)	
Others	18 (2.6)	12 (2.4)	
Age (yr), *mean ± SD*	62.2 ± 13.2	66.3 ± 15.3	**<0.001**
Age group, *n* (*%*)			
< 40	35 (5.1)	23 (4.5)	<0.0001*
40–49	82 (11.9)	45 (8.9)	
50–59	177 (25.7)	106 (20.9)	
60–69	180 (26.2)	109 (21.5)	
70–79	149 (21.7)	116 (22.8)	
> 80	65 (9.5)	109 (21.5)	
GCS on admission^#^, *n* (*%*)			
Mild	363 (53.4)	232 (45.8)	0.486
Moderate	153 (22.6)	135 (26.7)	
Severe	164 (24.1)	140 (27.6)	

^1^Statistical tests: ethnic and age group, chi-square test; age, 2-sample t-test

*Statistical significance due to gender disparity in >80 group.

^#^Mild GCS impairment is defined as GCS >12, moderate impairment as 9–12 and severe as ≤ 8

**Table 2:** Clinical characteristics at admission by gender. Females had lower eye and verbal components of GCS, overall GCS, and diastolic blood pressure on admission when compared to males. There was otherwise no difference between the genders for clot size, clot location, and background cerebrovascular risk factors (including smoking history, hypertension, diabetes, hyperlipidemia, statin, warfarin and anti-platelet use).

Parameters	Male (n = 688)	Female (n = 508)	P-value^1^
**Admission parameters**			
GCS, *mean ± SD*			
Eye	3.23 ± 1.2 (n = 680)	2.95 ± 1.27 (n = 507)	**0.016**
Verbal	3.24 ± 3.10 (n = 680)	3.00 ± 2.84 (n = 507)	**0.022**
Motor	5.07 ± 1.52 (n = 680)	4.96 ± 1.52 (n = 507)	0.201
Overall	11.4 ± 4.04 (n = 680)	10.9 ± 4.03 (507)	**0.030**
Fixed pupils, *n* (*%*)	63 (9.2) (n = 683)	51 (10.0) (n = 508)	0.636
Blood/Pulse pressure, *mean ± SD*			
SBP	180.1 ± 33.6 (n = 524)	180.9 ± 37.2 (n = 409)	0.732
DBP	98.3 ± 21.0 (n = 522)	93.1 ± 21.3 (n = 408)	**< 0.0001**
**CT scan characteristics**			
Clot size (cm^3^)	29.1 *±* 40.0 (n = 636)	26.1 *±* 22.7 (n = 481)	0.216
Clot location, *n* (*%*)			0.548
Deep	456 (66.3)	319 (63.2)	
Lobar	98 (14.2)	84 (16.6)	
Brainstem	53 (7.7)	37 (7.3)	
Cerebellar	55 (8.0)	45 (8.9)	
Intraventricular	16 (2.3)	8 (1.6)	
Multiple clots	10 (1.5)	12 (2.4)	
Hydrocephalus, *n* (*%*)	188 (27.3)	127 (25.0)	0.369
**Background cerebrovascular risk factors, *n* (*%*)**			
Hypertension	538 (40.9)	374 (39.1)	0.548
Diabetes	170 (12.9)	131 (13.7)	
Hyperlipidaemia	293 (22.3)	219 (22.9)	
Statin use	137 (10.4)	108 (11.3)	
Warfarin	40 (3.0)	39 (4.1)	
Anti-platelet agent	139 (10.6)	85 (8.9)	

^1^Chi-square test for categorical variables, 2-sample t-test for continuous variable

**Fig 1:**
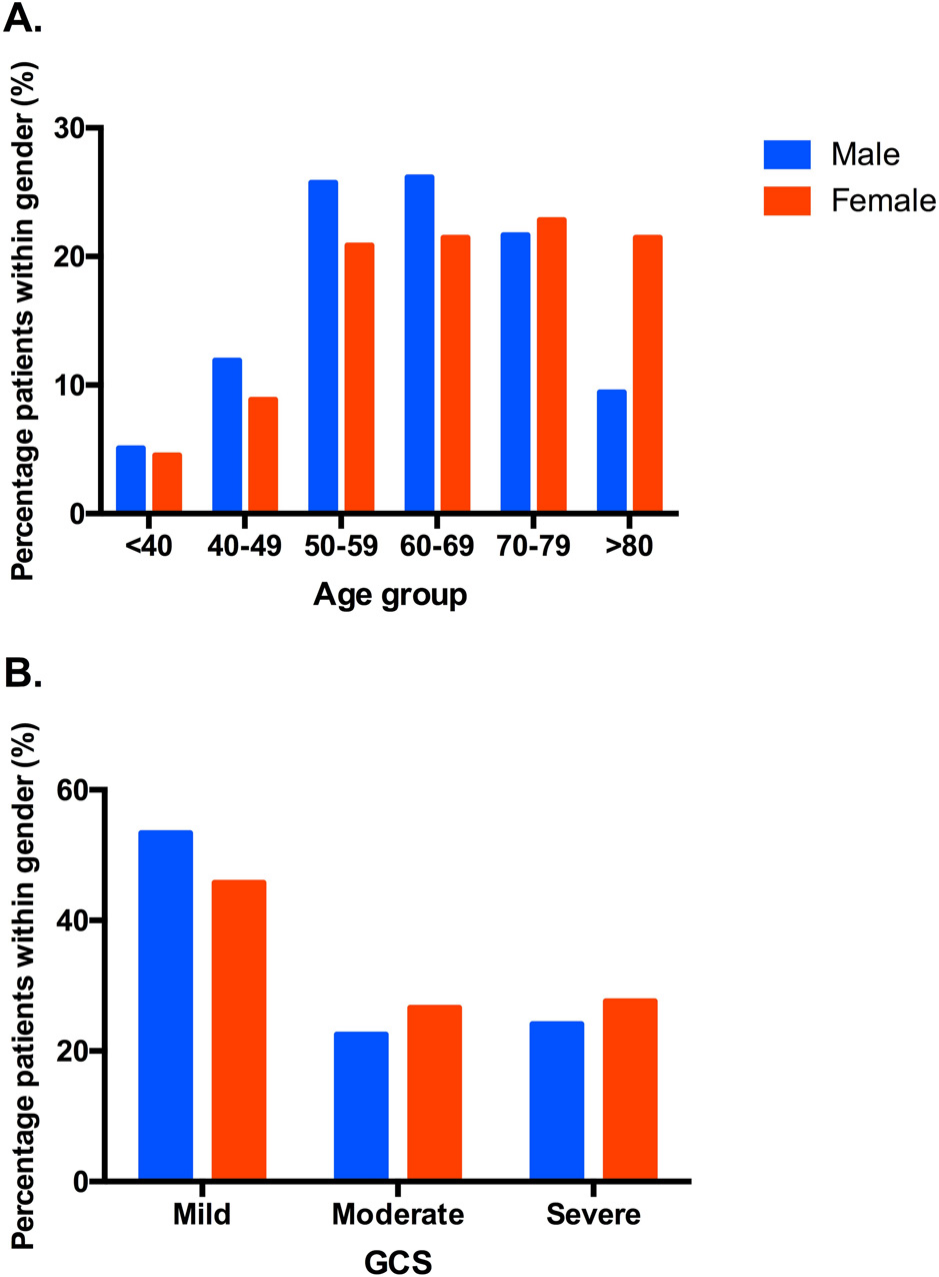
Percentage of patients with ICH by age and Glasgow Coma Scale (GCS) severity categories in relation to gender at admission. (A) Males were more likely to get ICH than females in all age groups, except for the age group >80 years. (B) There were no statistically significant gender differences when GCS was categorized into mild, moderate, and severe. Mild GCS impairment is defined as GCS >12, moderate impairment as 9–12 and severe as ≤ 8.

At admission, there were no statistically significant differences in clot size, clot location, cerebrovascular risk factors (smoking history, hypertension, diabetes, hyperlipidemia, statin, warfarin and antiplatelet use), and categorized GCS between genders. GCS on admission differed between males and females relative to eye, verbal and overall (P<0.05), but not motor (P = 0.201) scores (Table 2). Diastolic blood pressure on admission was significantly higher in males versus females (98.3 ± 21.0 vs. 93.1 ± 21.3, P<0.001; Table 2). Significant confounders identified via the stepwise selection algorithm were GCS on admission, age, clot volume, warfarin use, anti-platelet drug use, fixed pupils on presentation, clot location and hydrocephalus. There were no statistically significant differences between genders for the CT scan parameters (clot size, clot location profile, hydrocephalus) or the background cardiovascular risk factor profile. The percentage of patients with ICH by age category and gender at admission is plotted in Fig 1A. The percentage of patients in each GCS severity category by gender at admission is plotted in Fig 1B.

**Fig 2:**
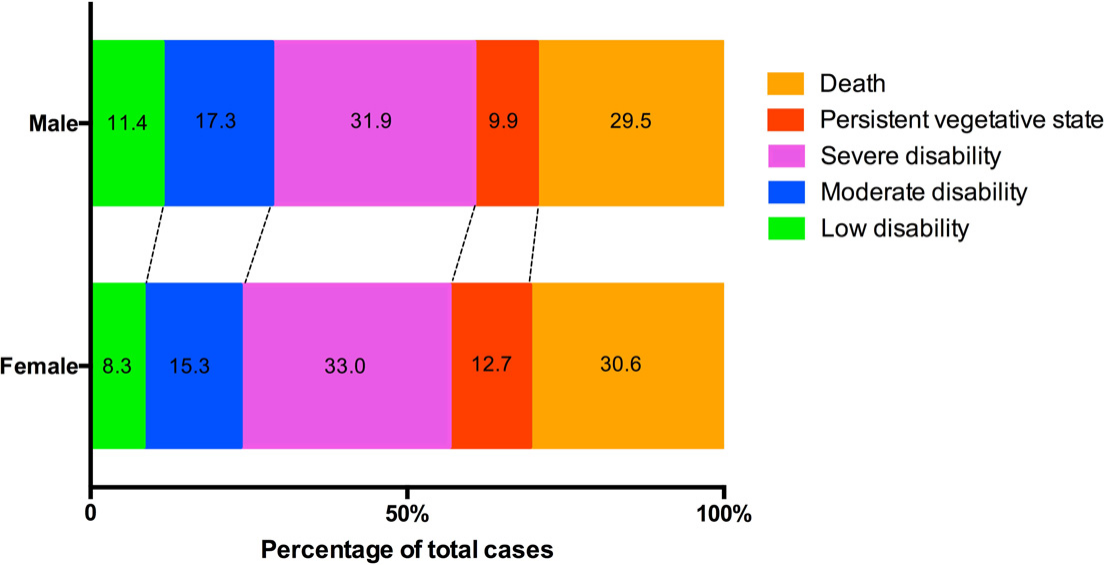
Glasgow Outcome Scale (GOS) on discharge. Differences between the genders for GOS on discharge were not statistically significant. However, females exhibited a trend of poorer GOS scores that could be clinically relevant, even though our study was not powered to detect significance.

The effects of gender on patient outcomes following ICH are summarized in Table 3. The difference in 30-day mortality rate between males (27.0%) and females (30.5%) was not statistically significant (p = 0.188) (AOR = 1.21; 95% CI, 0.84–1.75). As measured by the dichotomized GOS (4–5 vs. 1–3), females exhibited a slightly worse functional outcome on discharge (23.7%) compared to males (28.7%) but the difference was not statistically significant (P = 0.434) (Adjusted odds ratio = 0.87; 95% CI, 0.62–1.23). Our study was not powered to detect this difference in GOS-measured morbidity. A graphical comparison of the GOS between males and females at discharge is given in Fig 2.

**Table 3:** Effects of gender on patient outcomes following intracranial haemorrhage. There were no differences, even after adjustment, for 30-day mortality and dichotomized GOS score between the genders.

Outcome Variables	Male (n = 688)	Female (n = 508)	Unadjusted OR	P-value	Adjusted OR^2^	P-value
Mortality (%)						
≤ 30-days (%)	27.0 (186/688)	30.5 (155/508)	1.19^a^ (0.92, 1.53)	0.188^†^	1.21^a^ (0.84, 1.75)	0.300^†^
Glasgow Outcome Scale Dichotomized GOS						
GOS 4–5 (%)	28.7 (194/677)	23.7 (119/503)	0.77^a^ (0.59, 1.00)	0.055^†^	0.87^a^ (0.62, 1.23)	0.434^†^
GOS 1–3 (%)	71.3	76.3	—	—	—	—
GOS, *mean ± SD*	2.71 *±* 1.35 (n = 677)	2.58 *±* 1.29 (n = 503)	Unadj. Diff. 0.13 (-0.02, 0.28)	0.096^**‡**^	Adj. Diff. ^1^^,^^2^ 0.03 (-0.08, 0.15)	0.569^††^

^†^ Wald chi-square from logistic regression

^‡^ two-sample t-test

^††^Analysis of covariance

^1^Adjusted for gender, GCS on admission, warfarin, anti-platelet use, fixed pupils on presentation, clot location, hydrocephalus, age and volume of clot

^2^ Least squares means: Male, 2.16; Female, 2.13

^a^ Odds ratio calculated as Females/Males

## Discussion

While the effect of gender on several acute brain injuries (including ischemic stroke, traumatic brain injury, and spinal cord injury) have been well established, less is known about ICH [12–15]. In this relatively large cohort of Southeast Asian ICH patients, we showed that males were more susceptible to ICH than women for all groups under 80 years old. This is consistent with studies from other regions of the world including Europe, Australia, and Japan [6, 16–20]. Beyond 80 years of age, occurrence of ICH in female patients increased. Contrary to findings in the Western population, lowered mortality in females was not demonstrated in our study population [7, 8, 14, 21]. Age as an effect modifier on gender in determining ICH outcome is consistent with a previous study in a North American context [10].

While ICH occurrences were lower in females, neurologic symptoms on admission were more severe, as demonstrated by lower admission GCS score. This phenomenon was not associated with any specific haemorrhage location or background cerebrovascular risk. It was, however, associated with lower diastolic blood pressure on admission in female patients; the cause and consequence of this observation with regards to ICH progression remain unclear. This early neurological deficit was associated with a statistically non-significant, but clinically intriguing GOS trend towards greater morbidity in women at discharge. Our study was not powered to detect this difference in GOS-measured morbidity. There were, however, no significant differences in mortality between the genders.

In addition to informing the impact of gender on ICH epidemiology and disease progression, our current study has a pertinent impact on therapeutic decision-making. There has been recent interest in female gonadal hormones as a neuroprotective agent, as they have demonstrated efficacy in ICH and several other pre-clinical models of neurological diseases, including traumatic brain injury, ischemic stroke, and subarachnoid hemorrhage [22–33]. In our population, morbidity was worse in females than males after ICH, congruent with previous studies of traumatic brain injury in the same population [14]. This finding is also consistent with several other smaller-scale reports of detrimental interaction between female gender and ICH in other Asian populations [34–37]. Together, the evidence suggests that female gonadal hormone is unlikely to be neuroprotective in the Asian population.

In contrast to the Asian population, several Western studies showed reduced morbidity and mortality in female ICH patients compared to their male counterparts [6–8, 10, 11]. The basis for this difference remains unclear and requires clarification. It is also interesting to note that females demonstrate more severe functional morbidity after ischemic stroke, similar to our findings in ICH [38–40]. That ICH and ischemic stroke, two neurological diseases with different underlying mechanisms, show a similar trend renders it worthwhile to further investigate the pathophysiologic interaction between female gender and stroke.

Taken together, findings from our current study suggest that in our Southeast Asian population, female gender, particularly in younger women, can reduce occurrence of ICH. However, once ICH has occurred, females tend to have poorer neurological status on admission and worse morbidity than males at discharge. The mechanisms underlying the current observations, however, remain unclear. Additional mechanistic studies to investigate the observed differences in pathophysiology between gender and ICH progression is warranted.

There are several limitations to the current study. One is that we need longer-term outcome of this large cohort of patients. The differences in ICH outcome between the genders may not become apparent until beyond discharge. Another limitation is that while we used GOS as an accepted functional outcome metric, we did not have other relevant scores such as National Institute of Health Stroke Scale (NIHSS) for additional correlation. Finally, due to the retrospective nature of the study, we were not able to measure relevant factors such as hematoma expansion, cerebral edema, and cerebral perfusion to correlate gender with the underlying pathophysiology of ICH.

## Conclusion

In our multi-ethnic Asian population, males were more susceptible to developing ICH than females at all ages other than those in the >80 year-old age group. In contrast to the Western population, neurological status of female ICH patients at admission was poorer and their 30-day mortality was not reduced. Although the study was not powered to detect significance, female showed a trend toward worse 30-day morbidity at discharge.

## Supporting Information

S1 Dataset Intracerebral Hemorrhage data set from National Neuroscience Institute, Singapore. (XLSX)
